# Impact of Diabetes Mellitus on Head and Neck Cancer Patients Undergoing Concurrent Chemoradiotherapy

**DOI:** 10.1038/s41598-020-64844-1

**Published:** 2020-05-07

**Authors:** Hsuan-Chih Kuo, Pei-Hung Chang, Cheng-Hsu Wang

**Affiliations:** Division of Hematology/Oncology, Department of Internal Medicine, Chang Gung Memorial Hospital, Keelung and Chang Gung University, College of Medicine, 200, Lane 208, Jijin 1st Road, Keelung, 20445 Taiwan

**Keywords:** Diabetes, Head and neck cancer

## Abstract

In this retrospective study, we investigated the impact of diabetes mellitus (DM) on patients with head and neck cancer (HNC) undergoing concurrent chemoradiotherapy (CCRT). We analyzed the demographic and clinical characteristics, treatment tolerance, and toxicities of patients with HNC undergoing primary or adjuvant CCRT with or without DM between 2007 and 2016. Of the 556 patients undergoing CCRT, 84 (15.1%) had DM. Compared with patients without DM, patients with DM were significantly older (56.2 ± 11.2 vs. 51.9 ± 9.5 years, P < 0.001), received lower doses of cisplatin (adjuvant CCRT: 175.30 ± 84.03 vs. 214.88 ± 68.25, P = 0.014; primary CCRT: 142.84 ± 79.49 vs. 187.83 ± 76.19, P < 0.001), and experienced higher rates of infection (adjuvant CCRT: 52% vs. 30.5%, P = 0.042; primary CCRT: 45.8% vs. 22.9%, P < 0.001). Among patients undergoing primary CCRT, compared with those without DM, the patients with DM experienced significantly higher rates of hematologic toxicity (65.7% vs. 39.3%, P = 0.004) and treatment-related deaths (10.2% vs. 3.5%, P = 0.051); and a greater weight loss (−6.17 ± 9.27% vs. −4.49 ± 6.84, P = 0.078). Patients with HNC and DM undergoing CCRT, compared with patients without DM, experienced higher rates of infection and hematotoxicity, loss of body weight, and higher treatment-related mortality.

## Introduction

Diabetes mellitus (DM) is a chronic disease affecting >8.5% adults worldwide^1^. It is associated with many complications, such as, retinopathy, nephropathy, neuropathy, and cardiovascular disease; DM has also been found to be strongly associated with carcinogenesis^2,3^. In Taiwan, head and neck cancer (HNC) is one of the five leading causes of cancer mortality; a strong male predominance has been reported^4^. The association between DM and HNC has been explored in several studies^5–7^. As a frequent comorbidity of cancer patients, DM is also reported to compromise the outcome of cancer treatment^8^. In a retrospective study using data from the American Nationwide Inpatient Sample database to investigate the postoperative impact of DM on patients with HNC, Raikundalia *et al*.^9^ found that compared with patients without DM, patients with DM undergoing surgery had significantly longer duration of hospitalization and higher rates of postoperative complications, such as postoperative infection and cardiac events. In contrast, a good glycemic control seemingly reduced the risk of postoperative complications in patients with HNC^10^. Besides surgical intervention combined with adjuvant radiotherapy with or without concurrent platinum-based chemotherapy, primary concurrent chemoradiotherapy (CCRT) is the mainstay of HNC treatment in patients with locoregionally advanced HNC. Additionslly, primary CCRT has been used for the treatment of HNC of the hypopharynx and oropharynx. Nonetheless, information focusing on the impact of DM on the patients with HNC undergoing concurrent chemoradiotherapy (CCRT) is yet to be determined. In this study, we aimed to assess the relationship between DM and the outcomes of CCRT in patients with HNC.

## Results

Of the 556 patients, 93.5% were male, 79.3% had locally advanced tumor stage IVA or IVB HNC, 84.0% smoked, 63.1% consumed alcohol, and 64.4% used betel quid (Table 1). No significant difference was found in the patient demographics between the DM and non-DM groups except for the mean age at HNC diagnosis (56.2 ± 11.2 vs. 51.9 ± 9.5 years, P < 0.001). There was an difference between the DM and non-DM group in T classification (P = 0.05), N classification (P = 0.05), and tumor site (P = 0.06), in which more T1/T2/T3 lesion, more N2 lesion, and more oral cavity lesion were found in the DM group, respectively. The treatment modality of the DM and non-DM groups was similar (primary CCRT: 70.2% in the DM group vs. 77.8% in the non-DM group, P = 0.13).

**Table 1:** Characteristics of DM and non-DM groups.

Characteristics	DM (n = 84)	Non-DM (n = 472)	*p* value
Age at HNC diagnosis (mean ± SD), years	56.2 ± 11.2	52.0 ± 9.5	**<0.001**
Male, n (%)	79 (94.0%)	441 (93.4%)	0.83
Body weight prior CCRT (mean ± SD), kg	62.2 ± 14.2	62.3 ± 12.6	0.61
Tumor stage, n (%)			0.66
I/II	4 (4.8%)	24 (5.1%)	
III	17 (20.2%)	70 (14.8%)	
IVA	48 (57.1%)	295 (62.5%)	
IVB	15 (17.9%)	83 (17.6%)	
T classification, n (%)			**0.05**
T1/T2	29 (34.9%)	156 (33.1%)	
T3	23 (27.7%)	82 (17.4%)	
T4	31 (37.3%)	233 (49.5%)	
N classification, n (%)			**0.05**
N0	15 (17.9%)	99 (21.0%)	
N1	22 (26.2%)	67 (14.2%)	
N2	42 (50.0%)	270 (57.2%)	
N3	5 (6.0%)	36 (7.6%)	
Tumor site, n (%)			0.06
Oral cavity	41 (48.8%)	175 (37.1%)	
Oropharnyx	18 (21.4%)	156 (33.0%)	
Hypopharnyx	25 (29.8%)	141 (29.9%)	
Treatment			0.13
Primary CCRT	59 (70.2%)	367 (77.8%)	
Adjuvant CCRT	25 (29.8%)	105 (22.2%)	
Exposure to smoking, n (%)	69 (82.1%)	398 (84.3%)	0.62
Exposure to alcohol, n (%)	56 (66.7%)	295 (62.5)	0.47
Exposure to betel quid, n (%)	53 (63.1%)	305 (64.6%)	0.79

Abbreviations: CCRT = concurrent chemoradiotherapy; DM = diabetes mellitus; HNC = head and neck cancer; SD = standard deviation.

Table 2 summarizes treatment tolerance and toxicities of adjuvant CCRT and primary CCRT in the DM and non-DM groups. The patients in the DM and non-DM groups undergoing adjuvant and primary CCRT received similar doses, fractions, and length of radiotherapy. For chemotherapy, compared with the non-DM group, the DM group received significantly lower accumulative doses of cisplatin in either adjuvant (175.30 ± 84.03 vs. 214.88 ± 68.25, P = 0.014) or primary CCRT (142.84 ± 79.49 vs. 187.83 ± 76.19, P < 0.001). The DM group also experienced significantly higher rates of infection (adjuvant CCRT group: 52.0% vs. 30.5%; primary CCRT group: 45.8% vs. 22.9%). Compared with patients without DM, a trend of greater weight loss was observed in patients with DM undergoing primary CCRT ( − 6.17 ± 9.27 vs. −4.49 ± 6.84, P = 0.078). In the primary CCRT group, compared with patients without DM, patients with DM experienced significantly higher rates of grade ≥3 hematotocixity (65.7% vs. 39.3%, P = 0.004) and treatment-related deaths (10.2% vs. 3.5%, P = 0.051). In contrast, patients with DM experienced significantly lower rates of grade ≥3 mucositis in the primary CCRT group (27.1% vs. 47.7%, P = 0.009).

**Table 2:** Comparison of treatment tolerance and outcomes between the DM and non-DM groups.

Variables	Adjuvant CCRT (n = 130)			Primary CCRT (n = 426)		
	DM n = 25	Non-DM n = 105	p value	DM n = 59	Non-DM n = 367	p value
Highest dose of RT received (mean ± SD), Gy	6272.00 ± 612.35	6196.00 ± 965.28	0.71	6907.59 ± 863.05	7043.44 ± 640.54	0.25
Number of RT treatment received with the highest radiation dose (mean ± SD)	32.70 ± 2.45	31.96 ± 3.53	0.38	34.66 ± 5.58	35.27 ± 2.68	0.56
Length of RT treatment (mean ± SD), weeks	7.51 ± 1.95	6.83 ± 1.32	0.065	7.42 ± 1.22	7.59 ± 1.29	0.51
CT CDDP dose (mean ± SD), mg/m^2^	175.30 ± 84.03	214.88 ± 68.25	**0.014**	142.84 ± 79.49	187.83 ± 76.19	**<0.001**
Weight loss during CCRT (mean ± SD), %	−6.48 ± 8.99	−3.33 ± 10.04	0.14^†^	−6.17 ± 9.27	−4.49 ± 6.84	**0.078**^**†**^
Neutropenic fever	20.0%	9.5%	0.26	11.9%	8.4%	0.39
Infection	52.0%	30.5%	**0.042**	45.8%	22.9%	**<0.001**
Grade ≥3 mucositis	28.0%	41.0%	0.45	27.1%	47.7%	**0.009**
Grade ≥3 pharyngitis	20.0%	20.0%	0.94	35.6%	49.3%	0.14
Grade ≥3 dermatitis	5.0%	7.1%	0.79	10.3%	12.8%	0.87
Grade ≥3 xerostomia	0.0%	1.2%	0.60	3.4%	1.2%	0.34
Grade ≥3 hematological toxicity*	47.6%	33.3%	0.22	65.7%	39.3%	**0.004**
Treatment-related death	8.0%	3.8%	0.71	10.2%	3.5%	**0.051**
1-year RFS rate	64.0%	67.6%	0.73	53.4%	66.4%	**0.055**
2-year RFS rate	48.0%	52.4%	0.69	46.6%	50.3%	0.60
1-year OS rate	80.0%	74.3%	0.55	60.3%	77.0%	**0.007**
2-year OS rate	52.0%	53.3%	0.90	48.3%	57.7%	0.18

Abbreviations: CDDP = Cisplatin; CT = chemotherapy; DM = Diabetes Mellitus; RT = Radiotherapy; SD = standard deviation; RFS = recurrence free survival; OS = overall survival.

^*^Including neutropenia, anemia, and thrombocytopenia.

^†^Nonparametric statistics, Mann–Whitney test.

**Figure 1:**
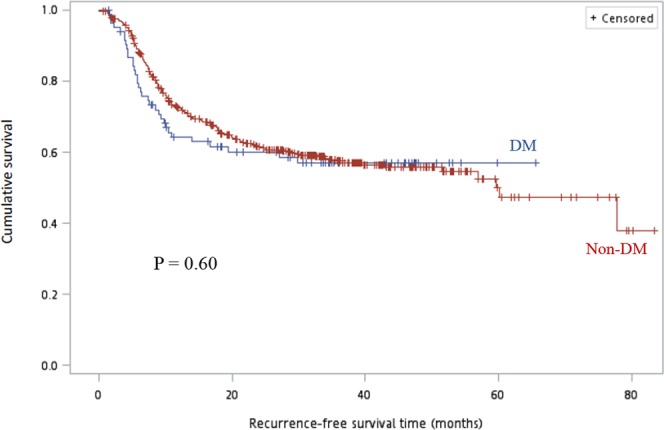
Comparison of recurrence-free survival (months) between DM and non-DM groups. Using the Kaplan–Meier method and the log-rank test, we did not detect a statistically significant difference between the groups, *P* = 0.60. Abbreviation: DM, diabetes mellitus.

**Figure 2:**
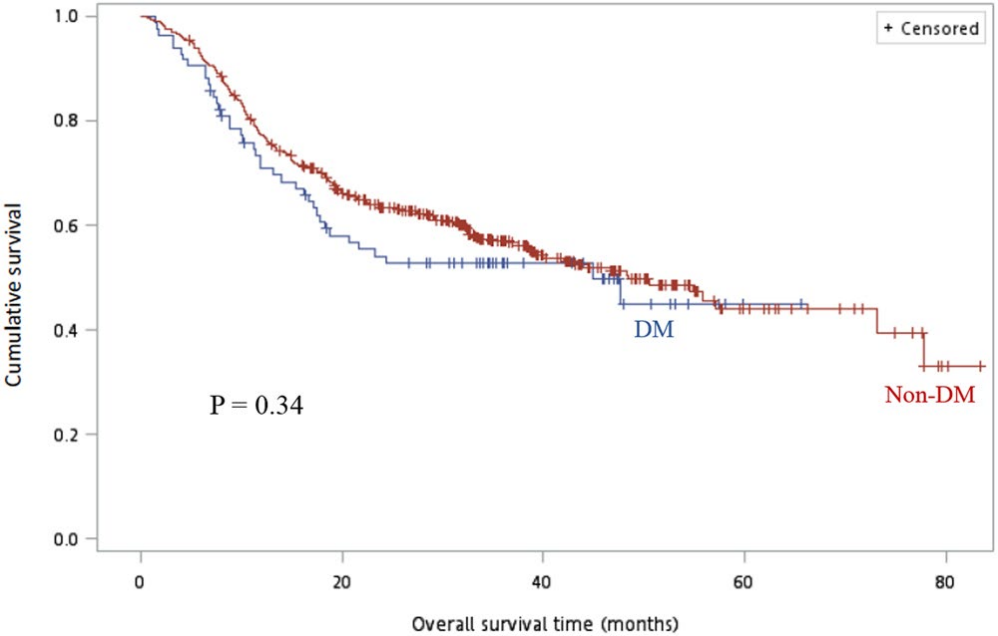
Comparison of overall survival (months) between the DM and non-DM groups. Using the Kaplan–Meier method and the log-rank test, we did not detect a statistically significant difference between the groups, *P* = 0.34. Abbreviation: DM, diabetes mellitus.

Regarding survival, in the adjuvant CCRT group, compared with non-DM patients, there were no significant differences in the RFS rate (64% vs. 67.6%, P = 0.73 and 48.0% vs. 52.4%, P = 0.69 for 1-year and 2-year RFS rate, respectively) and overall survival (OS) rate (80.0% vs. 74.3%, P = 0.55 and 52.0% vs. 53.3%, P = 0.90 for 1-year and 2-year OS rate, respectively). As shown in Table 2, compared with the non-DM group, the DM group showed a significantly lower 1-year RFS rate (53.4% vs. 66.4%, P = 0.055) and 1-year OS rate (60.3% vs. 77.0%, P = 0.007); however, the 2-year RFS rate (46.6% vs. 50.3%, P = 0.60) and 2-year OS rate (48.3% vs. 57.7%, P = 0.18) in the adjuvant CCRT setting was comparable. The RFS and OS curves for both the DM and non-DM groups are shown in Figs. 1 and 2, respectively and we found no significant differences (log rank P = 0.60 for RFS, P = 0.34 for OS) between the two groups. The multivariable Cox proportional-hazards models showed that tumor stage (HR = 1.75, 95% CI 0.78–3.93, P = 0.18; HR = 2.08, 0.97–4.45, P = 0.02; HR = 2.83, 95% CI 1.28–6.25, P = 0.01 for stage III, stage IVA, stage IVB, respectively, compared with stage I/II), doses of radiotherapy (HR = 0.98, CI 95% 0.96–0.99, P = 0.002), and infection (HR = 1.72 95% CI 1.32–2.24, P = 0.001) influenced survival independently (Table 3).

**Table 3:** Multivariable HRs for overall survival.

Variable	HR	95% CI of HR	P value
Age	1.00	0.99–1.01	0.93
DM	0.97	0.68–1.40	0.87
Stage*			0.02
Stage III	1.75	0.78–3.93	0.18
Stage IVA	2.08	0.97–4.45	0.06
Stage IVB	2.83	1.28–6.25	0.01
RT dose (Gy)	0.98	0.96–0.99	0.002
Cisplatin dose (mg/m^2^)	1.00	0.997–1.001	0.25
Weight loss during CCRT (%)	0.99	0.97–1.002	0.09
Infection	1.72	1.32–2.24	0.001

CCRT = concurrent chemoradiotherapy; CI = confidence interval; DM = diabetes mellitus; HR = hazard ratio.

*Reference category: stage I/II.

## Discussion

There is growing evidence showing that DM is an independent risk factor for cancer case fatality and all-cause mortality of cancer patients^3,8,11,12^. For locally advanced HNC, multimodality treatment, including surgery and CCRT, has been used for better tumor control. However, knowledge on the impact of DM on patients with HNC undergoing CCRT was limited.

The prevalence of DM in our studied population was 15.1%, which is comparable to those reported in other studies ranging from 12.8% to 18.5%^9,10,13,14^. In line with previous studies^9,10,14^, we also found that patients with HNC in the DM group were significantly older than those in the non-DM group. However, regardless of DM status, the mean age at HNC diagnosis in our studied was 9 to 13 years younger than other country^15^. This lower age at diagnosis of HNC in our study population could be due to the widespread use of betel quid, which is a Group I carcinogen listed by the International Agency for Research on Cancer that interacts synergistically with tobacco smoking and alcohol drinking and increases the risk of oral cancer to 123-fold^15,16^. Besides, betel nut chewing keeps increasing in male of younger generation^17^ and has been reported to be associated with type 2 DM in a prevalence study in Taiwan^16^, where betel quid chewing is endemic.

The tumor and the adverse effects of CCRT compromise the mastication and swallowing function often leading to weight loss of patients with HNC. Similarly, body weight changes are closely related to DM and different classes of DM medications have varied effects on body weight owing to their differences in gastrointestinal side effects or mechanism of actions^18,19^. In our study, in the primary CCRT group, we found that, compared with patients without DM, patients with DM patients experienced weight loss during CCRT. Weight loss is a poor prognostic factor implicated in worsening survival and increasing treatment-related complications in patients with HNC^20–22^. Therefore, nutritional intervention, such as nasogastric feeding tube or percutaneous gastrostomy before, during, and after CCRT should be implemented to attenuates weight loss and improve outcomes, especially in patients with DM.

DM has been associated with increased susceptibility to infections^23^. Therefore, it is not surprising that patients with DM and HNC experienced a significantly higher rate of infection compared with patients without DM and HNC undergoing surgery^9^. An increased risk of hematotoxicity in patients with DM patients receiving chemotherapy also predisposed them to infections because of cytotoxic agents suppressing the hematopoietic system, which further impaired the host protective mechanism^24^. Our study demonstrated that in patients with HNC receiving either adjuvant or primary CCRT, the presence of DM was related to significantly higher infection susceptibility. Additionally, patients with DM undergoing primary CCRT, experienced a significantly higher hematotoxicity than patients without DM. We found that both in the adjuvant and primary CCRT setting, patients with DM receiving significantly lower doses of cisplatin, which was more prominent in the primary CCRT group. It is most likely the higher infection and hematotoxicity rates influenced the doses of cisplatin given to patients with DM; consequently, these patients experienced less severe mucositis, which was also prominent in the primary CCRT group. In our study, we also found in the primary CCRT group, the patients with DM experienced significantly higher treatment-related death rates, and lower 1-year RFS/OS rates. Importantly, we found that infection during CCRT is an independent risk factor for OS and patients with diabetes were at high risk for infection. Because of significantly high risks of treatment-related complications, including infections, hematotoxicity, and death, the patients with diabetes and HNC undergoing CCRT, especially primary CCRT should be managed carefully.

It is well known that the diabetic patients have two-fold risks of death from macrovascular diseases^25^. Nonmalignant death of patients with HNC ranged between 15% and 35% at 5 years^26^. However, in the previous studies regarding survival, the effects of DM on HNC could not demonstrate consistent results. Ujpa’l *et al*. and Wu *et al*. found that DM had negative impact on the survival of patients with HNC undergoing multimodality treatment, including surgery, chemotherapy, and radiotherapy^27,28^. In contrast, Foreman *et al*. showed that DM alone did not adversely affect cancer survival outcomes of patients with HNC^14^. Besides, Spratt *et al*. found no difference in locoregional control between patients with and without DM and with oropharyngeal cancer^29^. In our study, compared with non-DM, patients with DM were older, experienced a higher infection rate, higher hematotoxicity, and more weight loss. After adjusting confounding factors, tumor stage, doses of radiotherapy and infection were independent risk factors for overall survival. However, the causes of deaths related to DM needs to be identified.

The limitations of our study include the relatively small size of patients, retrospective single center study design. We could not properly evaluate the duration of diabetes before cancer diagnosis and the severity of the disease. Besides, there are differences in the proportion for patients with oral cavity cancer and oropharyngeal cancer, which might cause different acute toxicity profile in response to radiation and lead to bias in reporting outcome. Tumor sites and treatment modality should be controlled in further prospective studies in order to confirm the study results. Nevertheless, our study is focused on the impact of diabetes on patients with head and neck cancer undergoing CCRT, which was not previously reported.

## Conclusion

Our study showed that DM significantly increased the treatment complication in terms of infection and hematotoxicity, leading to a greater weight loss, and caused a higher treatment-related mortality in patients with HNC patients undergoing CCRT. Patients with DM and HNC need more careful supportive care throughout CCRT period.

## Methods

### Patients

Data of patients with documented HNC who had undergone CCRT either as adjuvant or primary treatment were retrieved from the Chang Gung Memorial Hospital Cancer Registry (at the Keelung and Linkou campuses) between 2007 and 2016. Overall, 588 patients with HNC who had undergone CCRT were identified; of these, 32 were excluded due to drop-out from CCRT (3 patients), refusal to treatment (2 patients), had double or synchronous cancer (20 patients), had metastasis (2 patients), received RT alone (2 patients), repeatedly registered (1 patient), and transferred to other medical centers (2 patients). The remaining 556 eligible patients were then stratified into the DM or non-DM groups.

### Treatment and follow-up

All patients received intensity modulated or arc technique radiotherapy at a conventional fractionated daily dose of 180 or 200 cGy for five consecutive days per week. The total dose of radiotherapy was 7000–7400 cGy and 6000–6600 cGy for primary and adjuvant treatment, respectively. The initial treatment volume included the tumor bed and regional lymphatics. After receiving 4600–5000 cGy, the treatment area was reduced to the tumor bed and the regional nodes. The chemotherapy regimen were administered concurrently with radiotherapy according to the treatment guideline at our institution: cisplatin 40 mg/m^2^ every 1 week, cisplatin 100 mg/m^2^ every 3 weeks, cisplatin 50 mg/m^2^ plus oral UFT capsule (tegafur plus uracil, 250 mg/m^2^/day) and oral calcium folinate (90 mg/day) on days 1–14 every 2 weeks, and cisplatin 60 mg/m^2^ on day 1 plus continuous infusion of 5-fluorouracil [FU] 800 mg/m^2^ on days 1–5, every 2 weeks. Treatment-related toxicities were graded according to the National Cancer Institute Common Terminology Criteria for Adverse Events, version 3.0. The infection events were defined as patients requiring hospital admission for intravenous antibiotics treatment.

### Study Outcomes

The demographic and clinical characteristics, including tumor stage and TNM classification upon diagnosis, doses of chemoradiotherapy received, rates of toxicities, recurrence-free survival rate, and overall survival rate of the DM and non-DM groups were analyzed. Recurrence-free survival (RFS) was defined as the time from the initiation of CCRT to the first evidence of recurrence of the primary tumor. Overall survival (OS) was defined as the time from the initiation of CCRT to death from any cause.

### Statistical Analysis

SAS statistical software version 9.4 (SAS Institute Inc) was used for the analyses. The association between groups and various clinical and pathological features in each group were investigated using the chi-square test or Fisher’s exact test (if the number in any cell was <5) for categorical variables. The differences in continuous and ordinal variables in demographic, doses of CRT, and treatment duration were determined using the two-tailed independent Student’s t-test. The normal distribution of these continuous variables was tested by Kolmogorov–Smirnov test. If these variables were not normally distributed, log transformation and the nonparametric Mann–Whitney test were used. We used the Kaplan–Meier method to estimate overall and progression-free survival; the log-rank test to ascertain the significance. Besides, Corresponding hazard ratios (HRs) were calculated with 95% confidence intervals (CIs) using the Cox proportional-hazards model. A P value <0.05 was considered significant (two-tailed).

### Ethics

This retrospective analysis was approved by the Institutional Review Board (IRB) of Chang Gung Memorial Hospital (IRB No: 201800998B0). The IRB waived the requirement of informed consent form for this study.
